# Unique dietary and oral hygiene behaviors in a cohort with clinically severe obesity: A cross sectional study

**DOI:** 10.1002/cre2.895

**Published:** 2024-05-10

**Authors:** Zanab Malik, Woosung Sohn, Shanika Nanayakkara, Kathryn Williams

**Affiliations:** ^1^ Faculty of Medicine and Health The University of Sydney School of Dentistry Surry Hills New South Wales Australia; ^2^ College of Health, Medicine and Wellbeing The University of Newcastle, School of Health Sciences (Oral Health) Ourimbah New South Wales Australia; ^3^ Nepean Blue Mountains Family Metabolic Health Service, Nepean Blue Mountains Local Health District Kingswood New South Wales Australia; ^4^ Charles Perkins Centre‐Nepean The University of Sydney Sydney New South Wales Australia

**Keywords:** clinically severe obesity, oral health, oral hygiene behaviors, unique dietary behaviors

## Abstract

**Background:**

An association between increased risk of dental caries with increased levels of clinically severe obesity has been reported. Data linking body mass index (BMI) and dietary behaviors, including at‐risk dietary factors and oral hygiene habits, are lacking in a cohort with clinically severe obesity. This study aimed to explore the dietary and oral hygiene behaviors in individuals with clinically severe obesity attending a hospital‐based obesity service.

**Methods:**

Adult patients attending a hospital‐based obesity service in Greater Western Sydney with clinically severe obesity were invited to participate in a self‐administered survey, which collected data on their nutritional and oral hygiene behaviors. Demographic data (age, gender) and BMI were extracted from the participants' medical records. The primary outcome was the relationship between BMI and frequency of ^toothbrushing.^

**Results:**

Of the 82 individuals who consented to participate, 81 (98.8%) completed the study questionnaire. The median BMI of the cohort was 49.1 kg/m^2^ (interquartile range [IQR]: 43.2–57.3 kg/m^2^) and median age 51 (IQR: 39–63) years. BMI was not significantly correlated with individual oral health behaviors (*p* > .05). Many participants reported dietary risk behaviors, which have the potential to influence their oral health.

**Conclusions:**

While oral health behaviors were not associated with increasing BMI, patients with clinically severe obesity in this study reported unique dietary behaviors and mixed oral hygiene habits that may complicate nutritional and dental management. Awareness of these behaviors among clinicians including dental professionals is required in this cohort.

## 1 INTRODUCTION

Obesity is recognized as a public health problem worldwide (WHO, 2000). The World Obesity Federation estimates that by 2025, 2.7 billion adults will be overweight, over 1 billion affected by obesity and 177 million will have severe obesity (Lobstein et al., 2022). The consequences of obesity include the development of physical and mental health problems, reduced mobility and disability and socioeconomic disadvantage (Dixon, 2010). These negative impacts worsen with increasing BMI. Clinically severe obesity can be defined as that which is complicated with serious medical and/or mental health complications and/or has risk of complications due to excess fat, in addition to other factors. This is typically more common at higher BMI and consistent with those individuals typically seen at a hospital‐based obesity service. Given the increased risk of medical comorbidities linked to obesity, it is imperative that people with clinically severe obesity, have adequate access to, and consistently utilize medical services, inclusive of oral healthcare services. As such, patients with clinically severe obesity are often considered for referral to Special Needs Dentistry departments for comprehensive dental management and for appropriate modifications to their dental treatment plan, including the use of bariatric dental chairs (Comyn et al., 2012; Geddis‐Regan et al., 2019; Malik, 2020; Reilly et al., 2009).

Some studies have reported poor oral health in people with obesity compared to the wider population without obesity (Östberg et al., 2012; Kantovitz et al., 2006; Marshall et al., 2016; Hayashi et al., 2022). There is a complex and non‐linear relationship between obesity and dental disease (Kantovitz et al., 2006; Marshall et al., 2016). A recently published study reported increased risk of dental caries with increased levels of BMI (Taghat et al., 2022). There is evidence of poor dental service utilization in those with clinically severe obesity (Malik et al., 2023). The increased awareness of oral health as an important component of general health and quality of life has been a focus of the World Health Organization (WHO) Global Oral Health Programme (Petersen, 2009).

There are clinical and service delivery implications of obesity for dentistry, such as associations of obesity with dental disease, the need for treatment planning modifications and requirement for specialized equipment (Marshall et al., 2016). Individuals with clinically severe obesity, such as those who may be seen at a hospital‐based obesity service, require further study to better understand their needs. This includes study of oral health behaviors, encompassing both oral hygiene behaviors and cariogenic dietary risk factors and behaviors, which may be more common or present differently amongst people with obesity.

In a cohort attending a public hospital‐based obesity service with clinically severe obesity, higher rates of adverse diet and physical activity behaviors and chronic disease were observed (Atlantis et al., 2018). Whether this group may similarly have poorer oral hygiene behaviors and be at greater risk for poor oral health needs to be determined. Individuals with clinically severe obesity may experience unique barriers to dental access, including poor accessibility of dental services, cost, fear and anxiety and not having a trusting relationship with the dental provider (Geddis‐Regan et al., 2019; Malik et al., 2021).

Despite the associations between obesity and dental disease, there is a paucity of research carried out amongst those with the most clinically severe obesity. Previous studies have used clinical indices and have focused on small sample sizes and lower BMI levels (up to BMI of 44.4kg/m^2^) (Forslund et al., 2002; Sede & Ehizele, 2014). There are minimal data on the link between increasing BMI and oral health behaviors in those with clinically severe obesity, who are potentially a group at highest risk for dental disease. Potentially modifiable at‐risk dietary behaviors relevant to individuals with clinically severe obesity also need to be explored. This study aimed to determine if adults attending a public hospital‐based obesity service with clinically severe obesity have poor oral hygiene habits, with a focus on their toothbrushing and flossing behaviors. The primary outcome was the relationship between BMI and frequency of toothbrushing. The dietary behaviors unique to this cohort were also reported.

## 2 MATERIALS AND METHODS

Between August 2019 and February 2020, all adult patients attending the Nepean Blue Mountains Family Metabolic Health Service (FMHS), a hospital‐based multidisciplinary obesity service, were invited to participate in the study. Due to limited resources and high demand, referral criteria for the FMHS requires patients to have both elevated BMI ( ≥ 40 kg/m^2^ minimum) *and* objective evidence of medical complications, or other high‐risk features (such as Type 2 diabetes mellitus, non‐alcoholic fatty liver disease with fibrosis, severe obstructive sleep apnea, prolonged hospital admissions due to obesity‐related conditions, mobility limitations, Aboriginal or Torres Strait Islander ethnicity or young age), unless BMI is ≥70 kg/m^2^. Participants were excluded if they were pregnant, aged <18 years of age, unable to provide informed consent to participate in the study and/or non‐English speaking.

This study was a single‐site project. Ethics approval was gained from the Nepean Blue Mountains Local Health District Human Research Ethics Committee and all participants provided informed written consent before participating. Study surveys were distributed in person, by electronic link or by mail for completion, depending on participant preference. Electronic surveys were sent via email through a database using the REDCap™ (Research Electronic Data Capture) web‐based software platform. All paper‐based and electronic survey responses were recorded and de‐identified in the REDCap™ database by a study investigator.

The survey questionnaire was informed by current literature and with expert advice from the FMHS clinicians, including dieticians experienced in the management of people with clinically severe obesity. It contained questions on oral hygiene practices, such as toothbrushing and flossing frequency, cariogenic dietary factors, including frequency of high sugar content foods and drink, as well as any specific dietary risk behaviors identified as an issue by clinicians for people with clinically severe obesity. These questions included “how often do you brush your teeth?”, “how often do you floss in between your teeth?”, “How often per day do you consume one serving of sugary foods? (one serving = a standard serving size on the food packaging label, for example, chocolate bar, handful of lollies, two biscuits, one slice of cake, etc.)”, “How often do you consume non diet or full sugar soft drinks/fizzy/sparkling drinks/juices? (one serving = 250 mL glass or 2/3 of a can)”. Participants were asked how often they consumed discretionary foods or drinks which are not required to meet nutrient requirements, typically including subgroups of foods and drinks higher in saturated fat, added sugar and/or salt (Hendrie et al., 2017). A list of food and drink examples was provided for reference. Participants could choose the following responses of “never or less than once per week”, “1–2 times per week”, “3–4 times per week”, “daily” or “more than once a day.” This last question was sourced from the CSIRO Healthy Diet Score Survey following consultation with department dietitians working with the study population as a good reflection of discretionary food or drink intake (Hendrie et al., 2017). The Commonwealth Scientific and Industrial Research Organization (CSIRO) Healthy Diet Score survey was not used in full to avoid participants experiencing “survey burnout.” These questions were asked either in person or by telephone, if they could not be sourced from the medical file.

Age and gender were recorded for each participant. The most recent height, weight and BMI for the pre‐existing patients of the service were extracted from the electronic medical records. In the circumstance when the new patients did not have a weight and height documented by the service, the height, weight and BMI were extracted from their referral documents.

**Figure 1:**
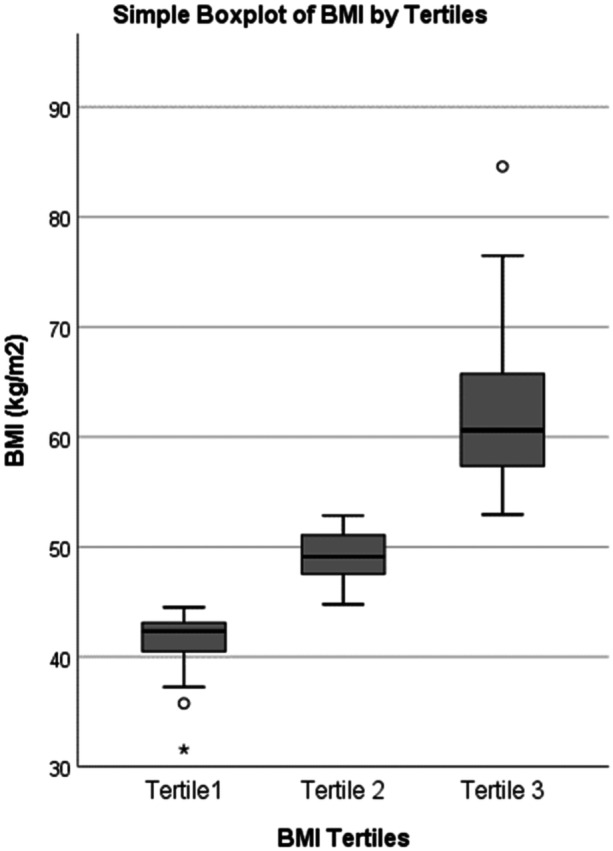
Distribution of body mass index (BMI) by tertiles.

Statistical analysis was performed using IBM SPSS Statistics (version 26, IBM SPSS Inc.). Data were presented as frequencies and percentages for categorical variables and median and interquartile range (IQR) for continuous variables due to the skewed distribution of data (see Figure 1). Data was represented in graphical form to assist interpretation, where appropriate.

**Figure 2:**
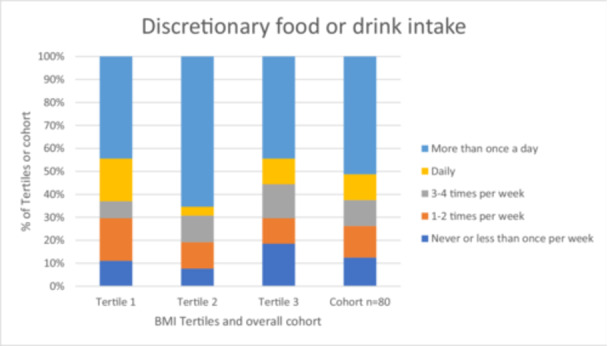
Participant responses to screening question on discretionary food or drink intake according to BMI tertiles and across the cohort (*n* = 80).

After performing graphical inspection of BMI data, the cohort was divided into BMI tertiles (see Figure 1). The age, gender, answers to the oral hygiene questions and dietary questions were described for the whole cohort and by BMI tertiles (see Figure 2).

Univariate associations between BMI tertiles and other study variables were determined using chi squared for categorical variables and Kruskal Wallis test for continuous variables. Spearman's rank correlation test was used to assess correlations between linear variables. The level of significance for all statistical tests was set at *p* ≤ .05. Data for dental risk dietary behaviors were described for the entire cohort only.

## 3 RESULTS

### 3.1 Clinical characteristics

Eighty‐two participants consented to participate in the study, however only 81 had completed the survey responses and only 80 completed the screening question on discretionary food or drink intake. During recruitment, one patient was excluded due to being <18 years old. Of the total participants, 57 (70.4%) were female; were predominantly middle aged and had a median BMI of 49.1 kg/m^2^ (IQR: 43.2–57.3 kg/m^2^) (Table 1). A majority of participants; 76 (92.6%) had Class III obesity (BMI of ≥40 kg/m^2^). Those in BMI Tertile 1 were significantly older than individuals in Tertile 3 (*p* = .02) (Table 1).

**Table 1:** Demographics of the study population by BMI tertiles.

Variable	Entire cohort *N* = 81	BMI tertile 1 *N* = 27	BMI tertile 2 *N* = 27	BMI Tertile 3 *N* = 27	*p* value
Age (years) (median, IQR±)	51 (39–63)	58 (45–68)	48 (35–64)	45 (37–58)	.02
Gender–male (number, % within group)	24 (29.6)	11 (40.1)	5 (18.5)	8 (29.6)	.20
BMI (kg/m^2^) (median, IQR)	49.1 (43.2–57.3)	42.3 (40.3–43.2)	49.1 (47.2–51.1)	60.6 (57.3–66.1)	<.05

Abbreviation: IQR, interquartile range

Oral hygiene practices of the study participants and cariogenic dietary factors are presented in Table 2. Dietary risk behaviors further explored by survey data amongst the 81 participants showed high frequencies of snacking/eating due to boredom and frequent snacking on sweet foods/drinks or binge eating (Table 3). Further breakdown per BMI tertile is available in Table 3.

**Table 2:** Oral hygiene and cariogenic dietary behaviors of the study population by BMI tertiles and entire cohort.

	Entire cohort *N* = 81 *n* (%)	BMI tertile 1 *N* = 27 *n* (%)	BMI tertile 2 *N* = 27 *n* (%)	BMI tertile 3 *N* = 27 *n* (%)	*p* value
**Oral hygiene behaviors**					
Toothbrushing irregularly (<1×/day)	17 (20.1)	6 (22.2)	5 (18.5)	6 (22.2)	.24
Flossing irregularly (<1×/day)	69 (85.2)	23 (85.2)	23 (85.2)	21 (77.8)	.74
**Cariogenic dietary factors**					
One serving of sugary food ≥1×/day	32 (39.5)	10 (37.0)	12 (44.4)	10 (37.0)	.37
One serving of full sugar soft drinks/fizzy/sparkling drinks/juices ≥1×/day	18 (22.2)	5 (18.5)	6 (22.2)	7 (25.9)	.99

**Table 3:** Dietary risk behaviors reported by the study population.

		Frequency (*n* = 81)	Percentage (%)
Constant hunger	Yes	27	33.3
	No	54	66.7
Forced vomiting or purging	Yes	4	4.9
	No	77	95.1
Regurgitation of food	Yes	12	14.8
	No	69	85.2
Boredom snacking/eating	Yes	60	74.1
	No	21	25.9
Waking up to eat in the night	Yes	12	14.8
	No	69	85.2
Taking medications with a sugary food or drink	Yes	17	21.0
	No	64	79.0
Frequent snacking on sweet foods or drinks/binge eating	Yes	47	58.0
	No	34	42.0

The correlation between increasing BMI groups and discretionary food or drink survey scores was presented in a graph format (see Figure 2). No significant difference was found between the BMI tertiles and discretionary food or drink intake survey scores (*p* > .05).

## 4 DISCUSSION

The National Study of Adult Oral Health 2017–2018 detailed the oral hygiene behaviors among Australian adults (Luzzi et al., 2020). Overall 96.3% of the Australian adult population reported toothbrushing at least once per day and 55.7% used dental floss in the previous week. These behaviors were associated with gender (higher proportion of females brushed with toothpaste at least daily), socioeconomic status and dental visiting patterns, with lower socioeconomic status associated with lower frequencies of toothbrushing and flossing (Luzzi et al., 2020). However, our cohort of people with clinically severe obesity had lower levels of regular toothbrushing and flossing behaviors compared to the general population. Of concern, 20.1% of the participants engaged in toothbrushing on a less than daily frequency.

The participants in this study showed high levels of irregular flossing frequency, which was consistent with the cross‐sectional survey results of Prpić et al., 2013 (Prpić et al., 2013). In the study by Prpic, participants with obesity (BMI ≥ 30 kg/m^2^) aged 31–75 years performed interdental cleaning less than participants with a BMI in the healthy or overweight ranges. However, the study included the use of interdental brushes in addition to flossing in their questioning (Prpić et al., 2013). Other studies have reported both a lower frequency of daily toothbrushing and flossing in individuals with obesity, however there was no indication of the severity of obesity in the study population for comparisons with this cohort to be made (Park et al., 2016).

The frequency of cariogenic dietary factors was low in this study population compared with Australian national averages. With regard to discretionary food intake, Australian Dietary Guidelines recommend occasional consumption in small quantities, with 0–3 serves per day typically suitable, adjusted by age, height and activity level (Brownie et al., 2015). Based on a survey of Australian adults in 2011–2012, an average consumption of 5–7 serves per day was reported (Australian Bureau of Statistics, 2011). In this cohort, only 51.2% reported discretionary food or drink intake of 1 or more serves per day. Whilst the lower rates in this cohort may have been due to participants' active involvement in a weight management clinic, this finding helps to dispel the common misconception of expecting poor dietary habits in all people with obesity (Chaput et al., 2014). In contrast, a much higher proportion of this cohort (22.2%) were consuming sugar sweetened drinks daily compared with national data given in 2017–2018, this was reported by 9.1% of adults aged 18 and over (Australian Bureau of Statistics, 2018).

The presence of dietary risk factor behaviors, such as forced vomiting or purging, regurgitation, binge eating and waking up in the night to eat was of particular note in this cohort with clinically severe obesity. Constant hunger was experienced by a large minority of participants, consistent with the biology of obesity, and this may also contribute to adverse eating behaviors. The literature has reported an increased prevalence of eating disorders among people with obesity, particularly binge eating disorder and night eating syndrome, however there is no widely available prevalence data of these behaviors in a specific population of people with clinically severe obesity in Australia (Lundgren et al., 2010; Mauro et al., 2008).

Another key finding of this study was that BMI was not significantly correlated with any individual oral health behaviors and indicators in this study population, suggesting that the reported increased risk of dental caries and BMI (Taghat et al., 2022) may be related to other factors than weight alone or that this relationship is not evident at more severe levels of obesity. This is consistent with the findings of the cross‐sectional study involving a population of dental outpatients in Nigeria by Sede & Ehizele, 2014 (Sede & Ehizele, 2014). Other studies which have examined the relationship between oral health indicators and obesity have reported the presence of numerous confounding variables and drivers of obesity. These may complicate associations between obesity and oral health, including lifestyle factors, dental anxiety, medical co‐morbidities and social determinants of health (Östberg et al., 2012). A “common risk factor approach” to oral disease prevention targeting social determinants of health has been recommended and is supported by current literature (Sheiham & Watt, 2000).

Obesity and dental caries share common risk factors, including eating behaviors, such as grazing, and diets which are high in fat, sugar and carbohydrates, ultra processed and high in energy (Östberg et al., 2012; Levine, 2012). Socioeconomic and lifestyle factors may be cofounding variables to the relationship between obesity and oral health including the use of preventive dental services (Östberg et al., 2012). A number of obesity‐associated systemic factors may contribute to dental caries risk, including malnutrition due to poor diet quality, which could result in salivary gland hypofunction and higher caries risk (Wijey et al., 2019; Cullinan, 2012). Pain or infection caused by untreated dental caries could also influence food intake by creating difficulty in chewing function. This may result in avoidance of particular foods, such as vegetables, overcooking of food, reducing nutrients, or preference for high‐sugar containing foods, leading to further weight gain (Sheiham, 2005; Basher et al., 2017). These factors can contribute to lower oral health related quality of life which has also been reported in a cohort with clinically severe obesity (Malik et al., 2023).

### 4.1 Study strengths and weaknesses

This study is, to the authors' knowledge, the first of this type to be carried out in Australia and worldwide. The advantage of this study was reporting on a cohort with clinically severe obesity, a group potentially less frequently encountered in a general dental and medical practice setting and in research. Recruitment from a non‐dental setting potentially reduced bias, in that participants were not already engaged with a dental service, and allowed for inclusion of participants from various backgrounds with differing dental experiences and beliefs. This study provides greater insight into some unique dietary risk factors and behaviors, which may have implications on diet history taking and the tailoring of dietary advice for people with obesity by dental professionals. It also brings awareness to the potential drivers for poor oral health in those with clinically severe obesity and understanding for why specialized bariatric dental facilities are needed.

This study was limited by the small convenience sample of 81 participants recruited from a single center. Participant ethnicity was not recorded and may have influenced results. Participants in this study may already have been engaged with lifestyle behavioral consultations with the various clinicians in the multidisciplinary hospital‐based obesity service, including dietitians, whilst others were new to the service. As such, some of the findings regarding cariogenic dietary factors and discretionary food/drink intake may have been under represented, particularly if positive behavioral changes were made before or during participation in this study. Furthermore, the study participants attending an obesity service may have been more motivated than those from the community with the same medical profiles. Additionally, survey‐based data may be perceived as subjective. Other studies previously carried out to investigate relationships between oral health variables were also of a cross‐sectional design and involved a clinical component (Östberg et al., 2012; Forslund et al., 2002; Sede & Ehizele, 2014).

Another limitation of the study may have been in survey design, with a number of questions using dichotomous variables of “yes” or “no”, which may not have accurately captured information pertaining to dietary risk factors and these may require further in‐depth investigation. More detailed assessment of eating behaviors would help determine if these were symptoms of other related conditions to obesity or independent eating behaviors. Survey questions pertaining to oral hygiene behaviors could have been enhanced to include other aspects such as mouthwash use or other methods of interdental cleaning. It would also have been beneficial to have questioned the timing of oral hygiene practices around specific dietary practices, such as if toothbrushing occurred after binge or night eating episodes.

Future studies should include clinical factors, a thorough dental examination to assess the clinical implications of oral hygiene and cariogenic dietary behaviors and assessment of social determinants of health and other recognized confounders identified in previous studies. This is of particular importance given the numerous barriers to accessing dental services that people with clinically severe obesity have reported in the literature (Malik et al., 2021).

## 5 CONCLUSIONS

Patients with clinical severe obesity attending a single center in Australia, reported higher levels of infrequent toothbrushing compared with national data, lower levels of cariogenic dietary factors and a range of dietary behaviors that pose risk to their oral health. Unique dietary risk behaviors may complicate dental management and awareness among dental professionals is required when assessing patients with clinically severe obesity. This study provides greater insight into some unique dietary risk factors and behaviors, which may have implications on diet history taking and the tailoring of dietary advice for people with obesity by dental professionals. No oral health behaviors were significantly associated with higher BMI in this cohort, suggesting that weight alone cannot be used as a factor to determine dental caries risk in clinically severe obesity. The study provides further evidence upon which to build an obesity‐specific oral care prevention program targeting oral health behaviors, with emphasis on the need to improve overall dental care and to assess for dietary behaviors that may contribute to poor dental health. The findings of this study serve as a foundation for further research using larger, multicenter and longitudinal study protocols to further assess at‐risk oral health behaviors across different populations with clinically severe obesity, ideally paired with clinical, socioeconomic and oral health examination data.

## AUTHOR CONTRIBUTIONS

Zanab Malik, Woosung Sohn, and Kathryn Williams conceived the study design and survey questions. Zanab Malik carried out data collection; Shanika Nanayakkara performed statistical analysis; and Zanab Malik, Woosung Sohn, Shanika Nanayakkara, and Kathryn Williams analyzed the data and were involved in writing the paper. All authors have approved the submitted and published versions of the manuscript.

## CONFLICT OF INTEREST STATEMENT

The authors Zanab Malik, Woosung Sohn, and Shanika Nanayakkara have nothing to disclose. Kathryn Williams reports grants, personal fees and nonfinancial support from Novo Nordisk, Pfizer and Lilly outside the submitted work, and is the Clinical Lead and Manager of the Nepean Blue Mountains Family Metabolic Health Service, a tertiary lifespan obesity service in Greater Western Sydney, New South Wales, Australia.

## Data Availability

The data that support the findings of this study are available from the corresponding author upon reasonable request.
